# Commentary: Interaction of the ADRB2 Gene Polymorphism with Childhood Trauma in Predicting Adult Symptoms of Posttraumatic Stress Disorder

**DOI:** 10.3389/fpsyt.2015.00136

**Published:** 2015-09-29

**Authors:** Udi E. Ghitza

**Affiliations:** ^1^Center for the Clinical Trials Network, National Institute on Drug Abuse, National Institutes of Health, Bethesda, MD, USA

**Keywords:** posttraumatic stress disorder, childhood, brain development, precision medicine, genetics, addiction, opioid-related disorders, substance use disorders

While posttraumatic stress disorder (PTSD) is relatively common among psychiatric conditions, it only emerges in a subpopulation of persons exposed to environmental trauma (1). Brain noradrenergic system dysfunction has been implicated in development of PTSD (2). However, unknown is the extent to which polymorphisms in brain noradrenergic system genes interact with childhood environmental trauma to alter resilience or vulnerability to developing PTSD in adulthood.

Using a cohort of predominantly male military veterans and another with predominantly female civilians, Liberzon et al. found that individuals with a beta2-adrenergic receptor (ADRB2) gene variant and childhood trauma are at heightened risk for adult PTSD (3). Interestingly, ADRB2 polymorphism has also been associated with a risk for the development of chronic pain (4). Another ADRB2 gene variant was associated with resilience to adult PTSD symptoms (3). Collectively, these findings suggest that ADRB2 gene variants affecting brain noradrenergic system function interact with childhood adversity to alter vulnerability or resilience to development of PTSD. Replication of this research in large cohorts is needed as well as physiological confirmation of abnormal noradrenergic system function (3).

Noradrenergic system dysfunction has also been implicated in increased susceptibility to stress-induced opioid use and craving in individuals with opioid use disorders (OUD) (5). Furthermore, PTSD frequently co-occurs with OUD (6, 7). Thus, research should prospectively investigate whether similar gene by childhood-adversity interactions heighten vulnerability to developing co-occurring PTSD and OUD.

One limitation of this study is insufficient information to determine whether it accounts for epigenetic and associated brain vulnerability factors from such childhood trauma that may also predispose to developing PTSD. It is difficult to determine whether this study controlled for pertinent non-specific neurobiological factors indirectly impacting response to trauma in a particular person, independent of this gene by environmental-adversity interaction. It is also unclear whether altered expression of this gene is tangential/incidental to co-occurring critical brain development changes altering PTSD vulnerability.

To substantively advance knowledge, longitudinal research with sufficient statistical power is needed using advances in neuropsychiatric imaging and bioinformatics to identify brain genetic variants, imaging, pharmacogenetic and other molecular biomarkers, and neuropsychiatric vulnerability factors interacting with traumatic environmental events in childhood to predict long-term departures from normal brain and cognitive development, associated with PTSD and/or chronic pain. Such research should also identify critical brain and cognitive development windows in prodromal stages where primary prevention approaches might particularly be targeted to prevent the onset of PTSD and co-occurring OUD. For such a study to be impactful, it should include a large heterogeneous sample representative of the population of interest, control for relevant developmental, socio-demographic, psychiatric, environmental, and family-history cofactors, and include validated patient-reported and clinician-measured outcomes of PTSD and OUD. Sampling designs using probability-based sampling of subgroups at greater risk for PTSD and OUD (e.g., positive family history, prenatal exposure to substances of abuse, externalizing psychopathology) may be needed to achieve the above objectives. A reasonable research approach may also incorporate genetically informative designs (for instance, family based) or subjects (for instance, twins, siblings).

To address these considerations, suitable research settings could be large healthcare system-based research networks containing existing electronic health record systems (EHRs) and virtual data warehouses, which routinely and in a consistent manner capture comprehensive longitudinal health data on a relevant population base of patients, which can be combined with genetic and environmental data from longitudinal-cohort research studies. Leveraging interoperable EHRs in such practice-based research networks can enhance recruitment time as well as allowing efficient and cost-efficient targeting of research subjects with relevant characteristics (8). Furthermore, standardized data collection, querying, extraction and storage procedures are needed to return clinical data in a consistent manner to a centralized repository and to permit semantic mapping to achieve health information interoperability. In addition, state-of-the-art data-collection procedures (for instance, computer-administered/assisted interviews), practices (for instance, cultural matching) and quality-control processes (for instance, random verification, logic-checking) should be used. Much could be learned using this “big-data science” approach in such research networks to identify critical gene by environment mediated development patterns of PTSD, co-occurring OUD, and remission, and efficiently targeting the above longitudinal research objective to relevant subgroups of patients. For such research to have potential for optimizing care tailored to specific patient subgroups (i.e., precision medicine), it should ultimately establish EHRs-based expert-defined, actionable clinical decision support (CDS) tools that could assist medical providers to identify and manage patient subgroups with PTSD, and common co-occurring conditions, such as chronic pain and opioid misuse/use disorder, in general medical settings. Actionable CDS tools for managing co-occurring PTSD and OUD should include validated screening and assessment tools, which could be utilized to help clinicians in the screening, identification, intervention or referral to treatment of PTSD and OUD in general medical settings, results of which can be incorporated into EHRs.

To conclude, to fill important precision-medicine research gaps described above, large practice-based research networks with standardized EHRs data collection, querying, extraction, and storage approaches are needed to collect and analyze relevant longitudinal data on large numbers of individuals with important phenotypic data to merge with genetic information. This could be efficiently accomplished by such systems having the facilities for, and expertise with, harmonized data collection and analysis of neuropsychiatric imaging data and genetic specimens for research, with accompanying patient-reported data routinely collected in clinical practice and EHRs/encounter data. For example, several practice-based research networks in the United States (9, 10) may be helpful, having extensive experience with using EHRs to identify, recruit, and consent suitable patients and providers in population-based samples, selected according to diagnosis, patient-reported measures collected in clinical practice, treatments received, utilization patterns, or socio-demographic characteristics. Conducting this type of big-data longitudinal research maximizes the potential to understand key brain vulnerability factors interacting with early childhood adversity to alter susceptibility to PTSD and co-occurring OUD. Knowledge of such information on neural mechanisms of PTSD and comorbid OUD is a precursor to developing patient-centered, precision-medicine approaches tailoring primary prevention and early intervention to high-risk patients who may benefit from them the most.

## Conflict of Interest Statement

The author declares that the research was conducted in the absence of any commercial or financial relationships that could be construed as a potential conflict of interest.
